# Author Correction: Membrane-binding and activation of LKB1 by phosphatidic acid is essential for development and tumour suppression

**DOI:** 10.1038/s41467-022-28923-3

**Published:** 2022-03-08

**Authors:** Giada Dogliotti, Lars Kullmann, Pratibha Dhumale, Christian Thiele, Olga Panichkina, Gudrun Mendl, Roland Houben, Sebastian Haferkamp, Andreas W. Püschel, Michael P. Krahn

**Affiliations:** 1 Molecular and Cellular Anatomy, University of Regensburg, Universitätsstr. 31, 93053 Regensburg, Germany; 2 Internal Medicine D, University Hospital of Münster, Domagkstr. 3, 48149 Münster, Germany; 3 Institute for Molecular Cell Biology, University of Münster, Schlossplatz 5, 48149 Münster, Germany; 4 Cells-in-Motion Cluster of Excellence, University of Münster, D-48149 Münster, Germany; 5 Department of Dermatology, Venereology and Allergology, University Hospital Würzburg, Josef-Schneider-Str. 2, 97080 Würzburg, Germany; 6 Institute of Dermatology, University Hospital Regensburg, Franz-Josef-Strauss-Allee 11, 93053 Regensburg, Germany

Correction to: *Nature Communications* 10.1038/ncomms15747, published online 26 June 2017.

This Article contains an error in Supplementary Fig. 6a. In Supplementary Fig. 6a the actin bands are inadvertently duplicated from the S6K bands.

The incorrect bands have now been replaced and are shown below.

In addition, this Article was published without a Supplementary Figure containing full blots for all of the blots shown in the Figures and Supplementary Figures. A figure containing the full blots, Supplementary Fig. 7, has been added to the appended Supplementary Information file.

## Supplementary information

new SI Fig 6a

old fSI Fig 6a

Supplementary Information

