# Supplementary material for: Membrane-binding and activation of LKB1 by phosphatidic acid is essential for development and tumour suppression
Source: Nat Commun. 2017 Jun 26;8:15747. doi: 10.1038/ncomms15747 (PMC5490199; doi:10.1038/ncomms15747)
Supplement: Supplementary Information — Supplementary Figures and Supplementary Reference [file ncomms15747-s1.pdf]

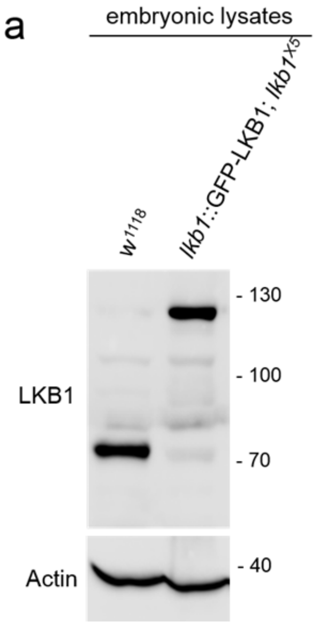


**Supplementary Figure 1: Expression of LKB1 by its endogenous promoter.**

(**a**) Western blot analysis Immunoblotting of embryonic lysates from $w^{1118}$ flies and homozygous mutants for $lkb1^{X5}$, an *lkb1* loss of function allele[1], that express GFP-LKB1 from the endogenous *lkb1* promoter shows comparable protein levels for endogenous and exogenous protein.

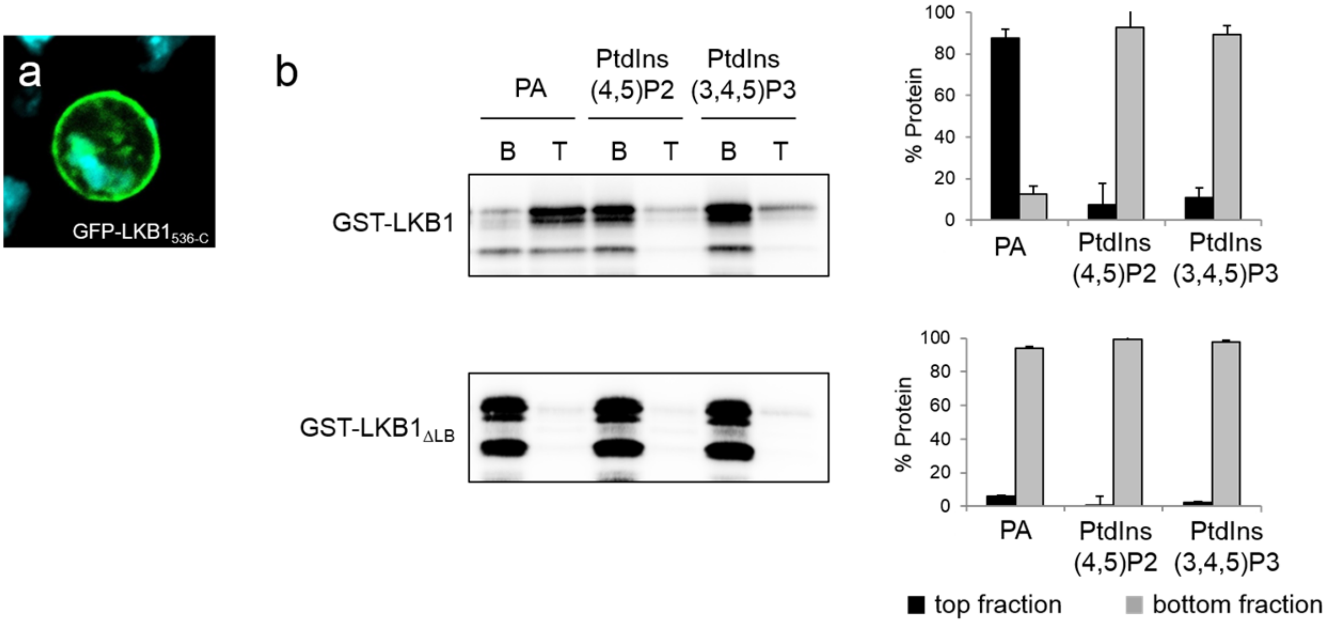


**Supplementary Figure 2: LKB1 binds to phosphatidic acid in liposome flotation assays.**

(**a**) GFP-LKB1$_{536\text{-}C}$ localizes to the cell cortex of transfected S2R cells. **(b)** Recombinant GST-LKB1 or GST-LKB1$_{\Delta LB}$ was incubated with liposomes (see Methods section for further details), overlaid with a sucrose cushion and subjected to ultracentrifugation. Liposomes were harvested and loaded as top fraction (T), whereas unbound protein remained in the bottom fraction (B). n = 3, significance (wild type LKB1 versus LKB1$_{\Delta LB}$): $p < 0.005$ for PA, n.s for PtdIns(4,5)P2 and $p < 0.05$ for PtdIns(3,4,5)P3.

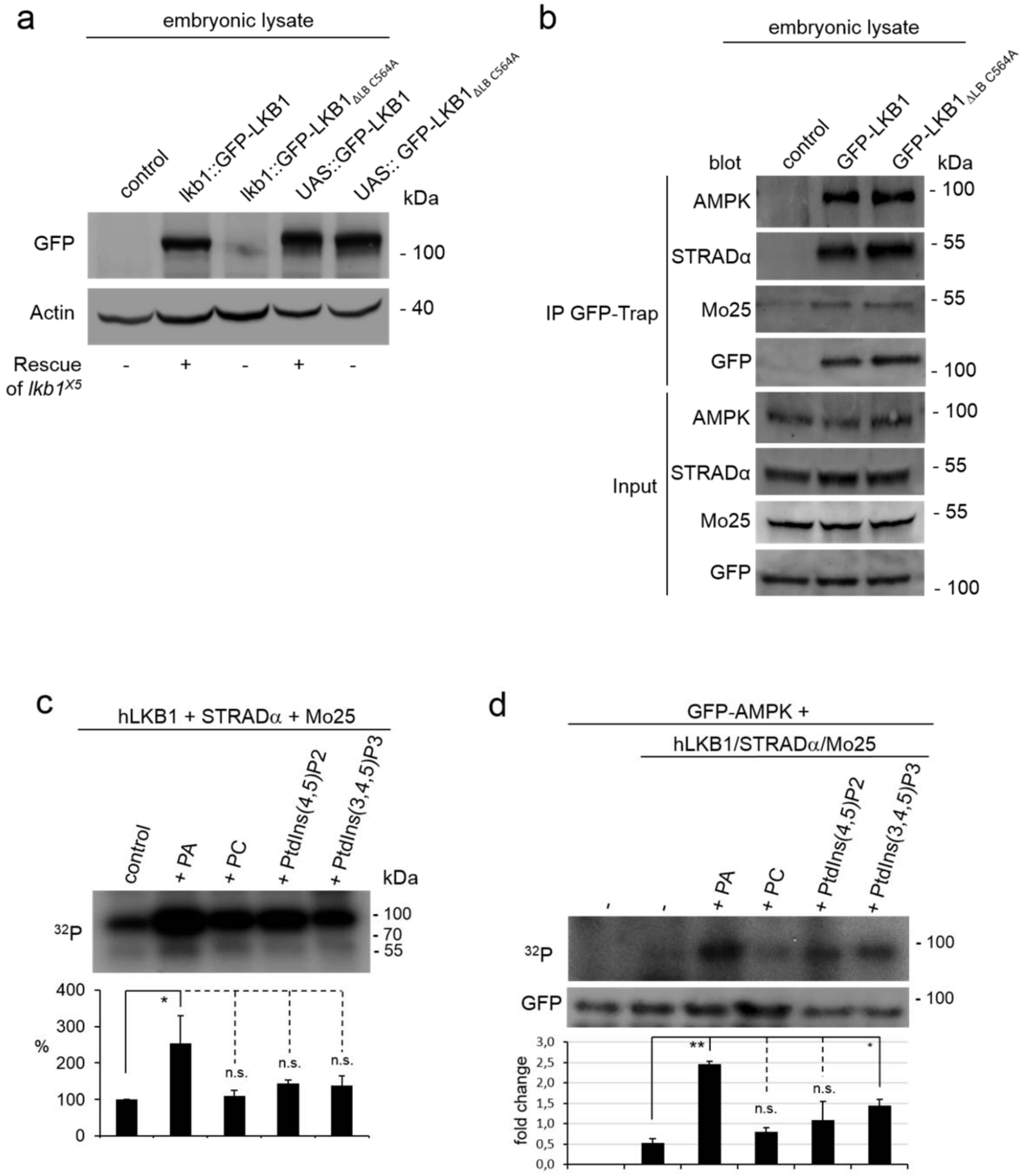


**Supplementary Figure 3: Lipid-binding of LKB1 is essential for its function.**

(**a**) Western Blotting of wild type or membrane binding deficient GFP-LKB1 expressed from its endogenous promoter (lkb1::GFP-LKB1) or ubiquitously expressed at endogenous levels using the UAS/GAL4 system (UAS::GFP-LKB1). Rescue capacity (viable flies: +) of these proteins in an *lkb1*$^{x5}$-mutant background is indicated. (**b**) Coimmunoprecipitation of GFP-LKB1 or GFP-LKB1$_{\Delta LB\ C564A}$ from embryonic lysates expressing GFP-LKB1 variants together

with HA-tagged STRADα and myc-tagged Mo25. Embryos expressing only HA-STRADα and myc-Mo25 but no GFP-LKB1 served as a control. (**c**) Kinase activity of recombinant hLKB1/hSTRADα/hMo25 is strongly increased by addition of PC + PA, but only slightly by PC, PC + PtdIns(4,5)P2 or PC + PtdIns (3,4,5)P3. Lipids were prepared as liposomes as described in methods sections and applied in a 9:1 (PC:X) molar ratio. (**d**) *In vitro* kinase assays of GFP-AMPKα1 purified from transfected HeLa cells using recombinant hLKB1/STRADα/Mo25 and indicated lipids. GFP-AMPK phosphorylation in the absence of LKB1 was not detectable. Phosphorylation of GFP-AMPK by LKB1 in the absence of lipids was set as 100%. Experiments were performed in triplicates. Error bars represent standard deviation and **s**tatistical significance was determined using ANOVA: $p < 0.0001$ ****, $p < 0.001$ ***, $p < 0.01$ **, $p < 0.05$ *, $p > 0.05$ not significant (n.s.).

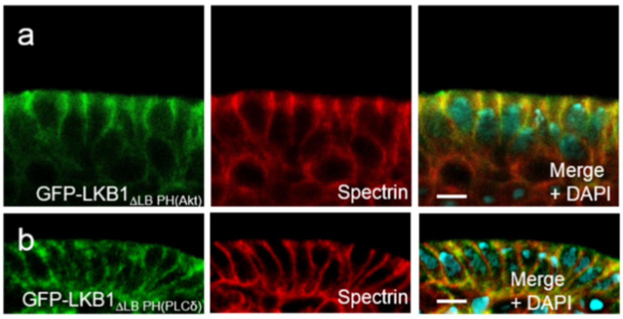


**Supplementary Figure 4: Localization of fusion proteins of GFP-LKB1$_{\Delta LB\ C564A}$ with heterologous membrane-binding domains.**

(**a** and **b**) Immunostainings of embryos with GFP and α-spectrin reveal a substantial cortical localization of GFP-LKB1$_{\Delta LB\ C564A}$ fused to the PH domain of Phospholipase C (PH(PLCδ), **a**) or to the PH domain of Akt1 (PH(Akt), **b**). Scale bars are 5µm.

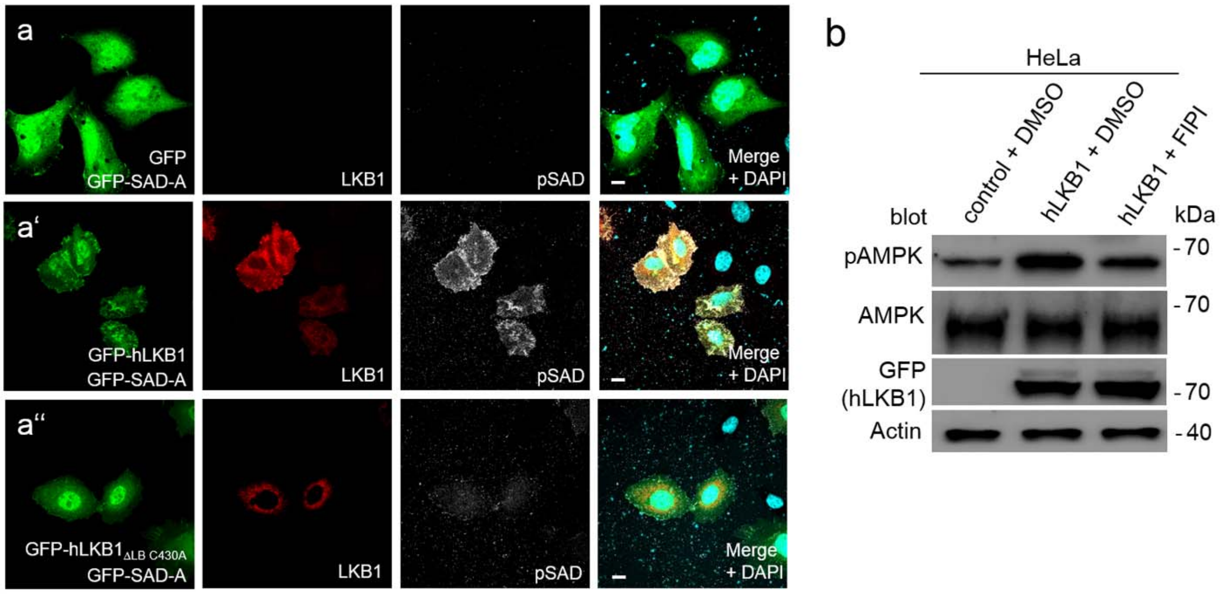


**Supplementary Figure 5: Activation of AMPK and AMPK-related kinases by LKB1.**

(**a**) HeLa cells were transfected with plasmids for GFP-SadA and GFP alone (**a**) or the indicated GFP-hLKB1 variants (**a'-a''**) and stained with antibodies specific for LKB1 (red) and activated Sad kinases (phosphorylated at S175 of SadA, pSAD, white). (**b**) Inhibition of PLD by 100nM 4-Fluoro-N-(2-(4-(5-fluoro-1H-indol-1-yl)piperidin-1-yl)ethyl)benzamide (FIPI) for 1h decreases the activation of AMPK by LKB1 in transfected HeLa cells. Scale bars are 5µm.

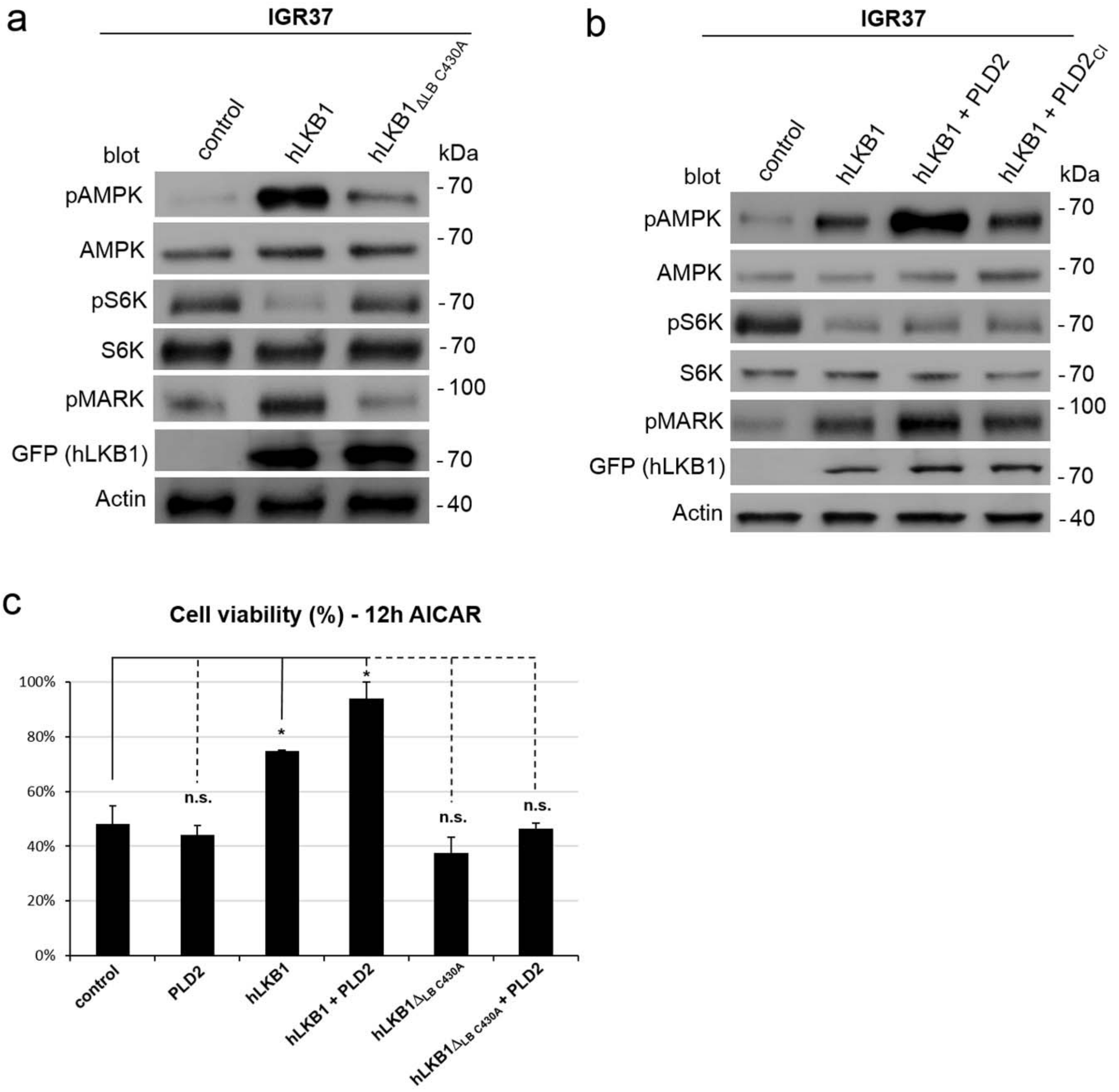


**Supplementary Figure 6: Activation of AMPK by LKB1 in malignant melanoma cells depends on membrane binding**

(**a** and **b**) The activation of AMPK and MARK kinases by LKB1 in was analyzed by Western blot in IGR37 cells, which lack detectable LKB1 expression. Upon transfection of hLKB1, AMPK and MARK are activated, which is not seen in a membrane binding deficient LKB1 variant. This activation is further increased in the hLKB1+PLD2 double transfected cells (**b**). (**c**) Cell viability of IGR37 cells under energetic stress (induced by incubation with AICAR for 12h) was estimated using the MTT assay (as described in the methods section). Experiments were performed in triplicates. Error bars represent standard deviation and **s**tatistical significance was determined using ANOVA: $p < 0.0001$ ****, $p < 0.001$ ***, $p < 0.01$ **, $p < 0.05$ *, $p > 0.05$ not significant (n.s.).

Supplementary Reference

1 Lee, J. H. *et al.* JNK pathway mediates apoptotic cell death induced by tumor suppressor LKB1 in Drosophila. *Cell Death Differ* **13**, 1110-1122, doi:4401790 [pii]

10.1038/sj.cdd.4401790 (2006).
