# Supplementary figures and images for: Author Correction: Membrane-binding and activation of LKB1 by phosphatidic acid is essential for development and tumour suppression
Source: Nat Commun. 2022 Mar 8;13:1283. doi: 10.1038/s41467-022-28923-3 (PMC8904824; doi:10.1038/s41467-022-28923-3)

**a****IGR37**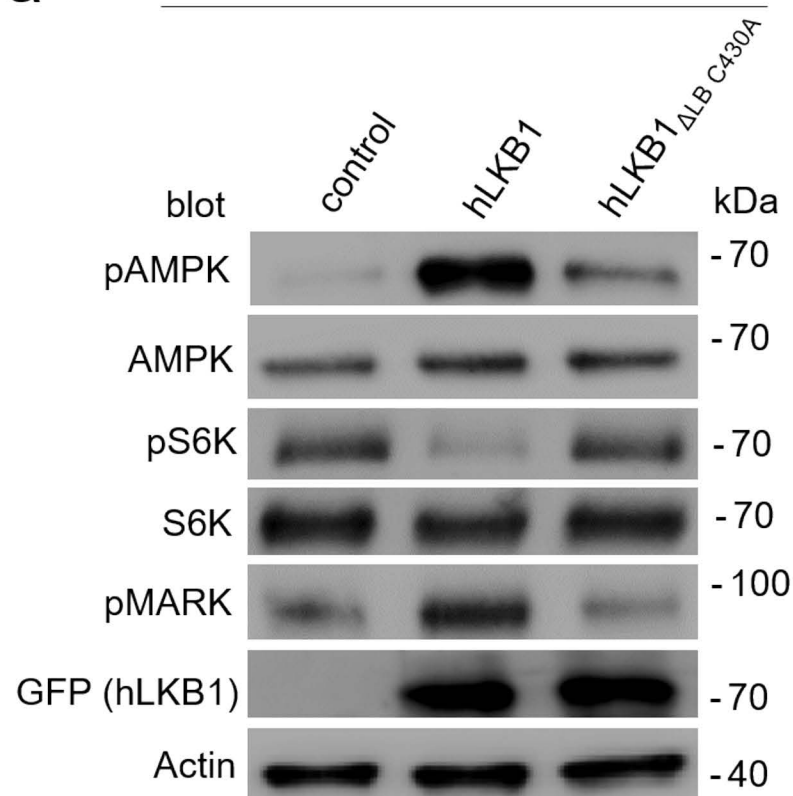**b****IGR37**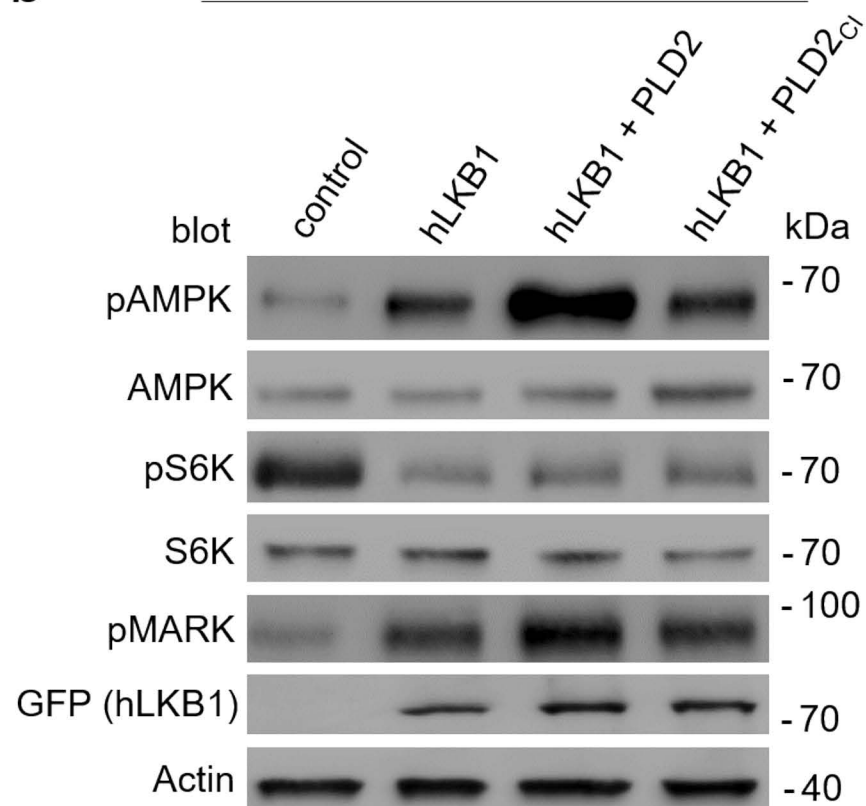**C****Cell viability (%) - 12h AICAR**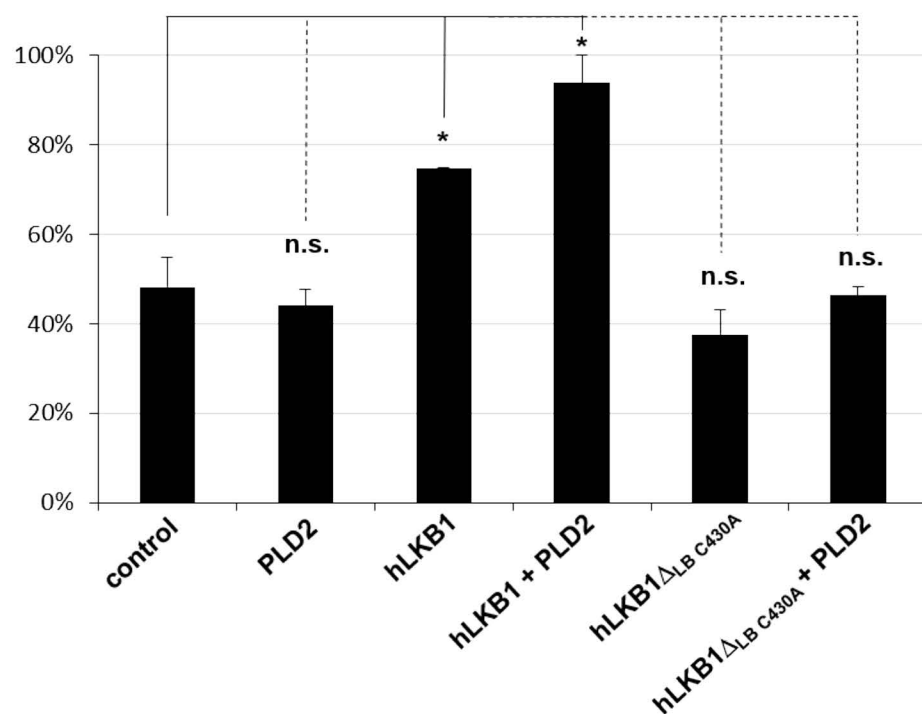

Supplement: Supplementary file 1 — new SI Fig 6a [file 41467_2022_28923_MOESM1_ESM.pdf]

Supplementary Figure 6

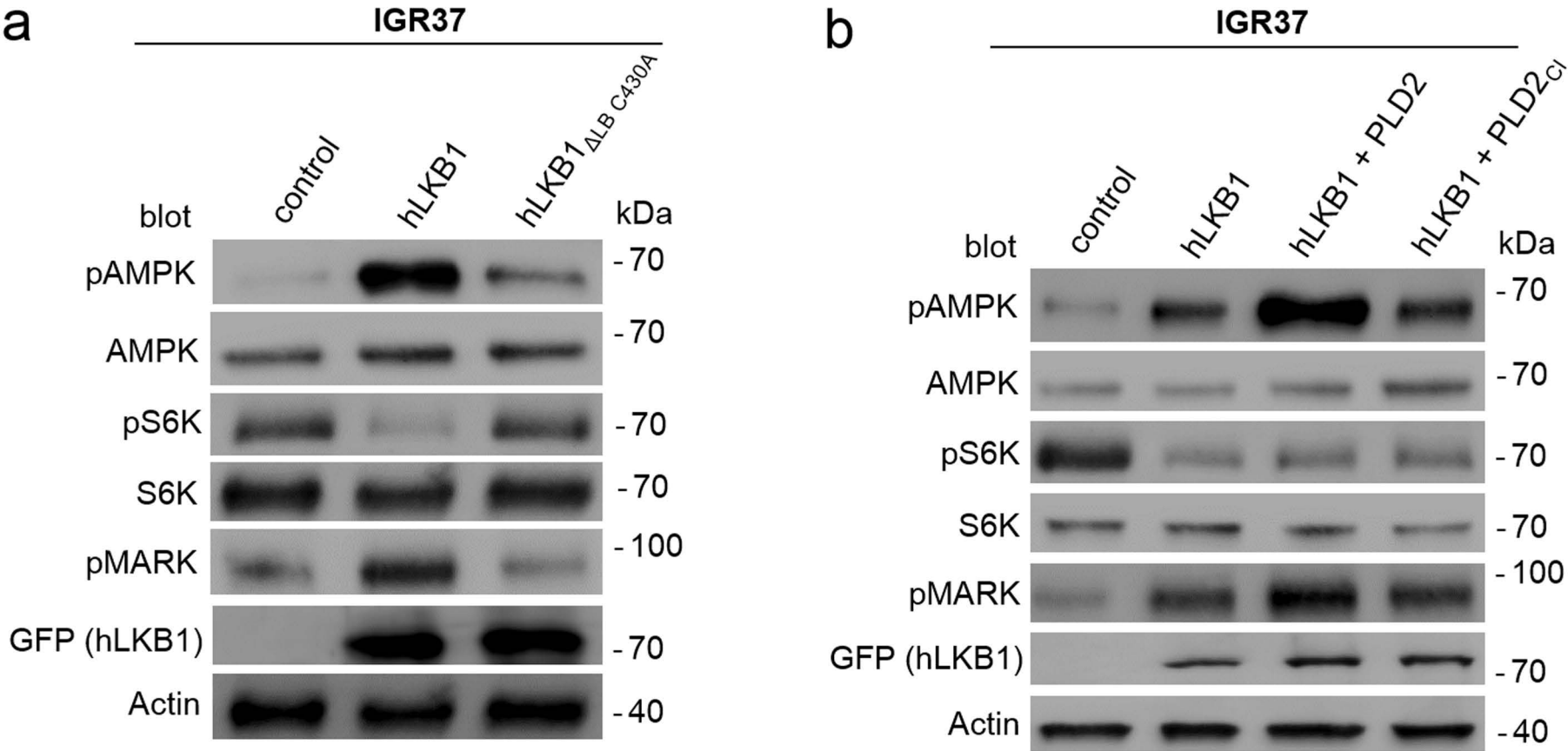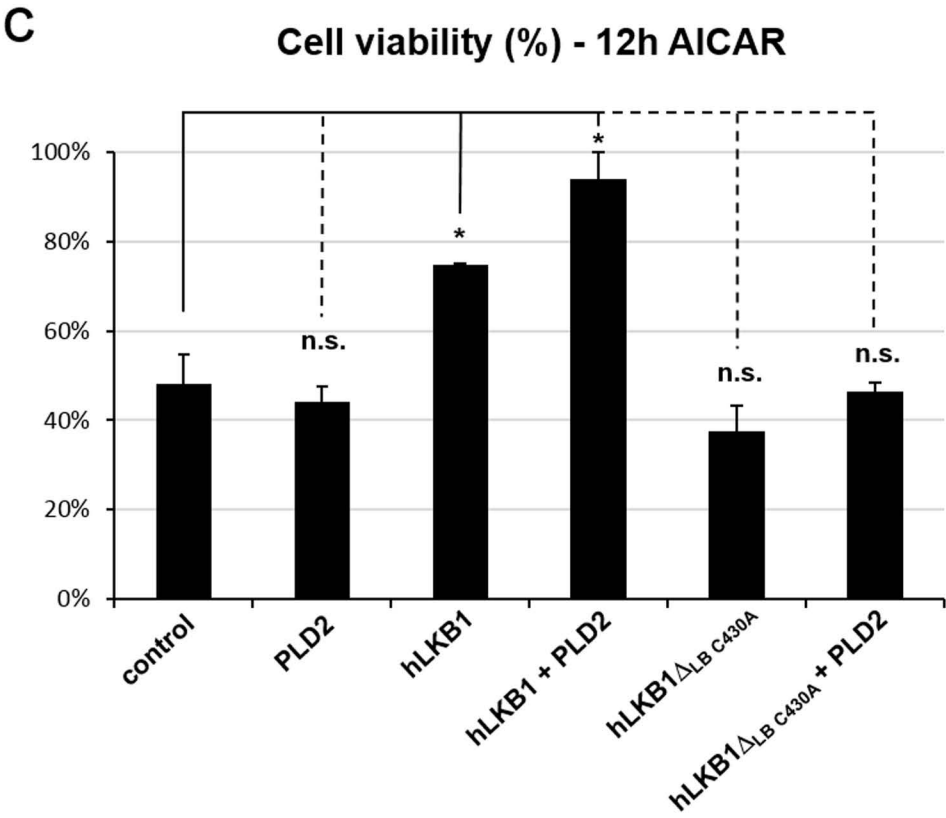

Supplement: Supplementary file 2 — old fSI Fig 6a [file 41467_2022_28923_MOESM2_ESM.pdf]
