# Supplementary material for: Author Correction: Membrane-binding and activation of LKB1 by phosphatidic acid is essential for development and tumour suppression
Source: Nat Commun. 2022 Mar 8;13:1283. doi: 10.1038/s41467-022-28923-3 (PMC8904824; doi:10.1038/s41467-022-28923-3)

Supplementary Figure 7 – original Western Blots

Fig. 2b GST-LKB1 blot

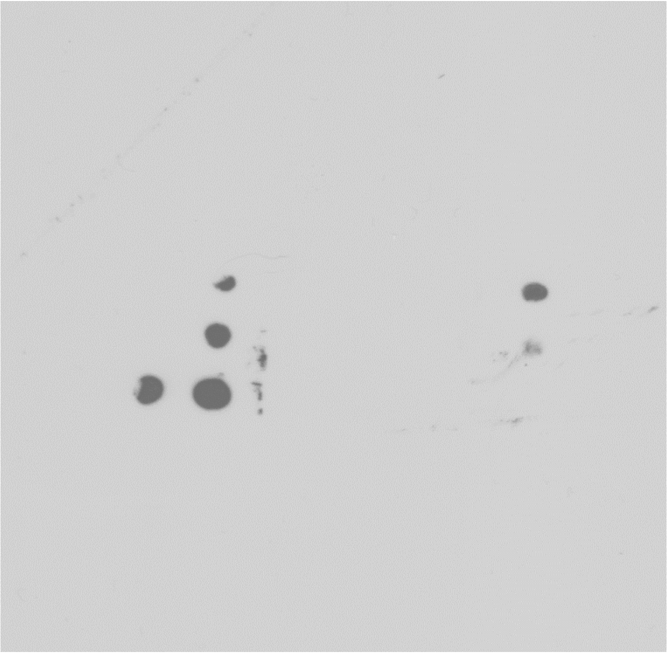

Figure 3d – GFP blot

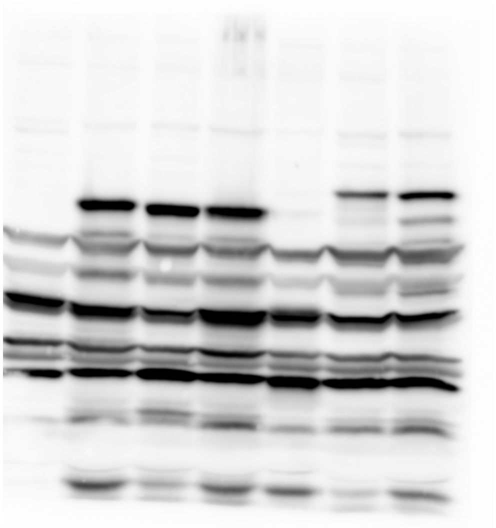

Actin Blot

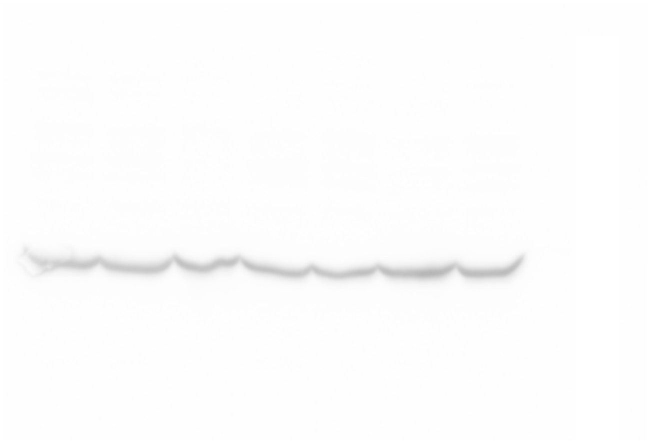

Figure 3f – GFP blot

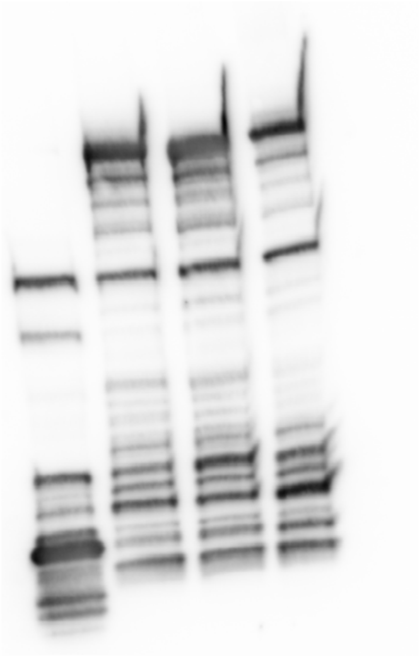

Figure 3g

GFP-Blot

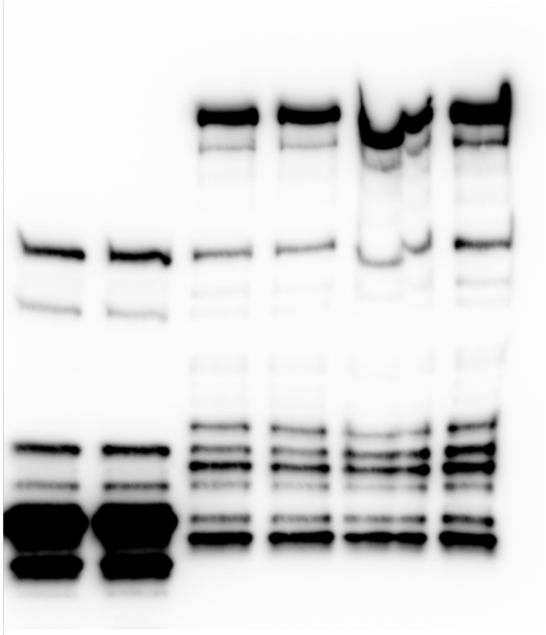

Figure 4d

pAMPK blot

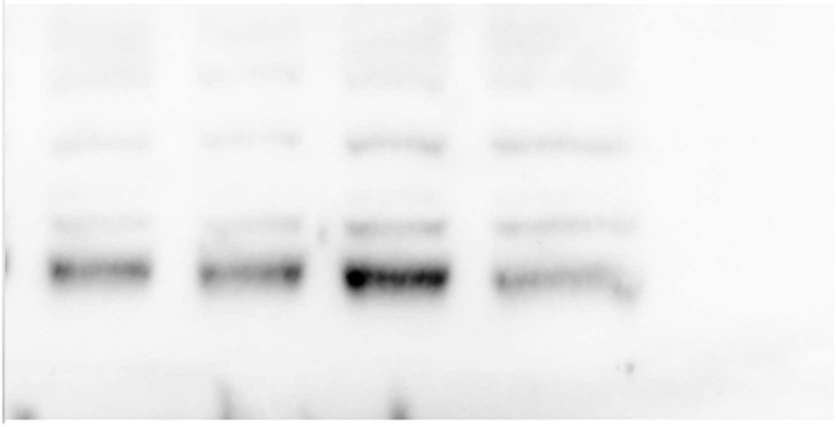

AMPK blot

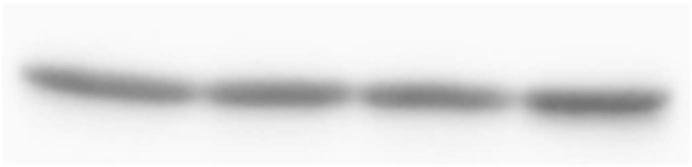

pS6K

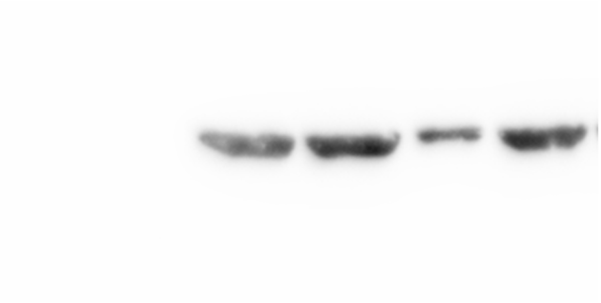

S6K

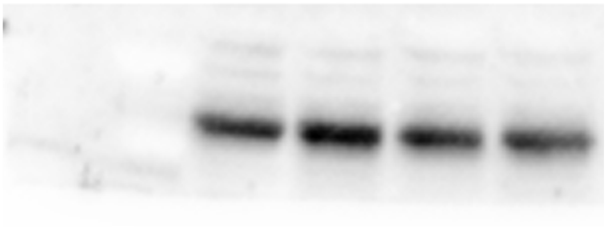

pMARK blot

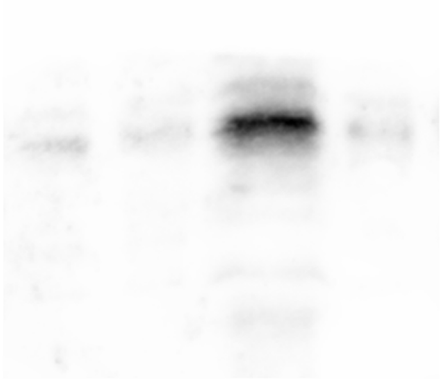

GFP blot

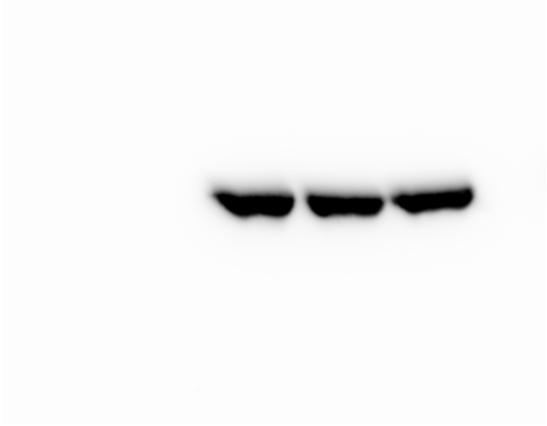

Actin blot

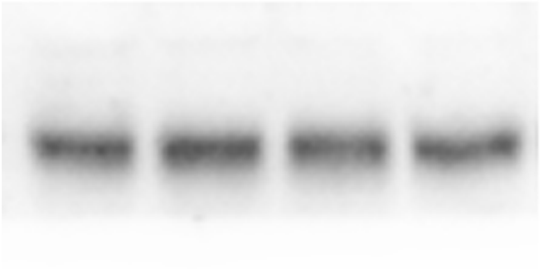

Figure 4e

pAMPK blot

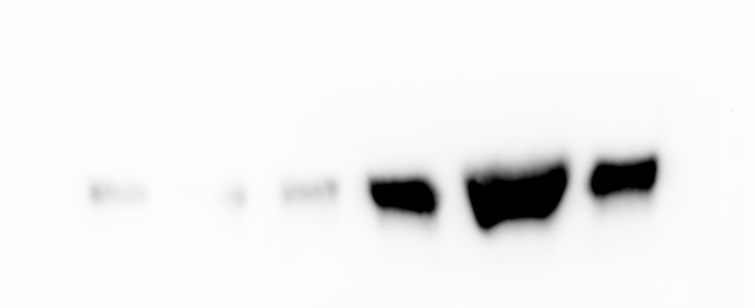

AMPK blot

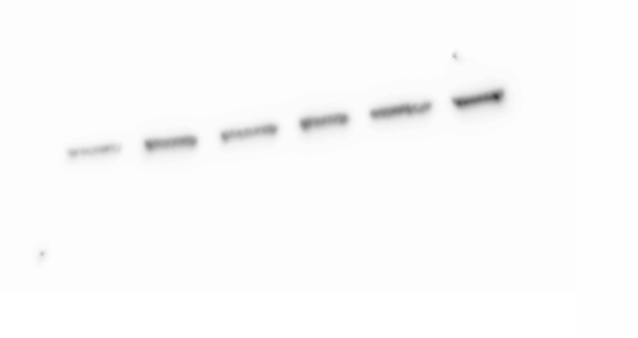

pS6 blot

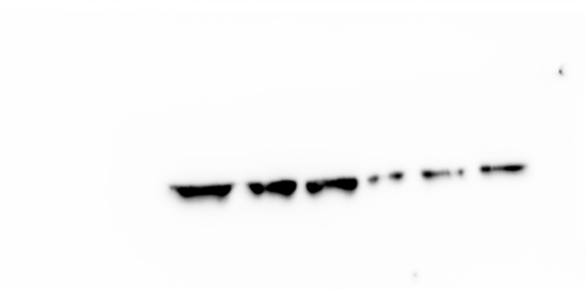

S6K blot

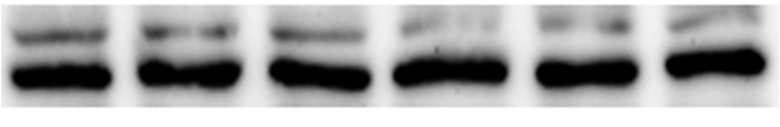

pMARK blot

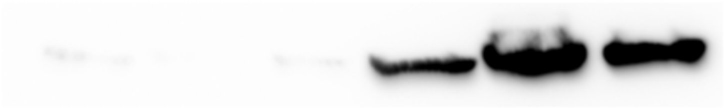

GFP blot

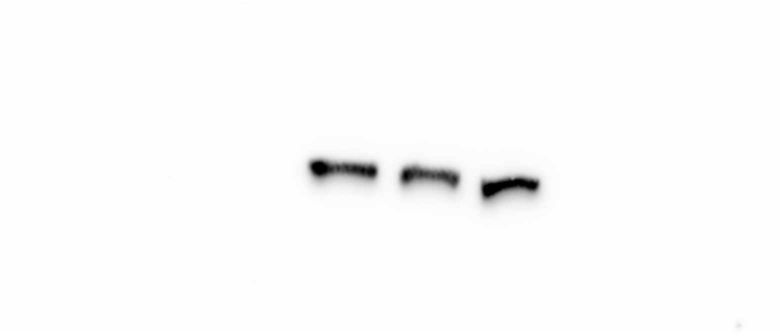

Actin blot

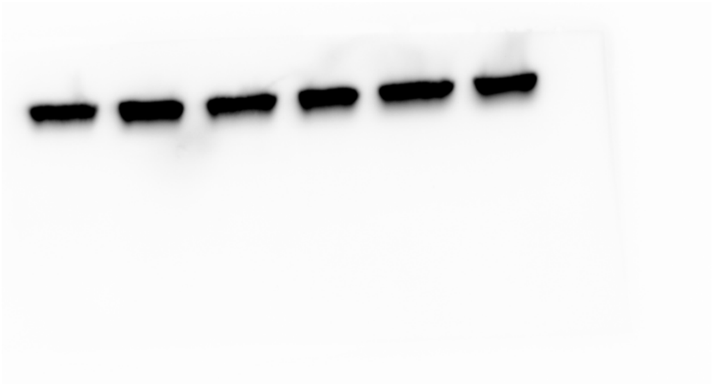

Supplementary Fig. 1a

LKB1 blot

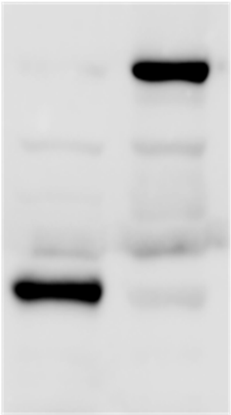

Actin blot (lane 1-2)

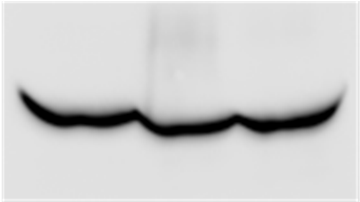

Supplementary Fig. 2b

GST-LKB1 blot (lane 3-8)

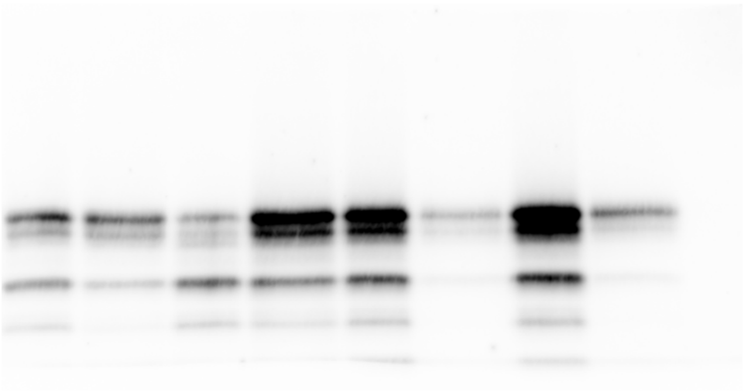

GST-LKB1ΔLB blot (lanes 3-8)

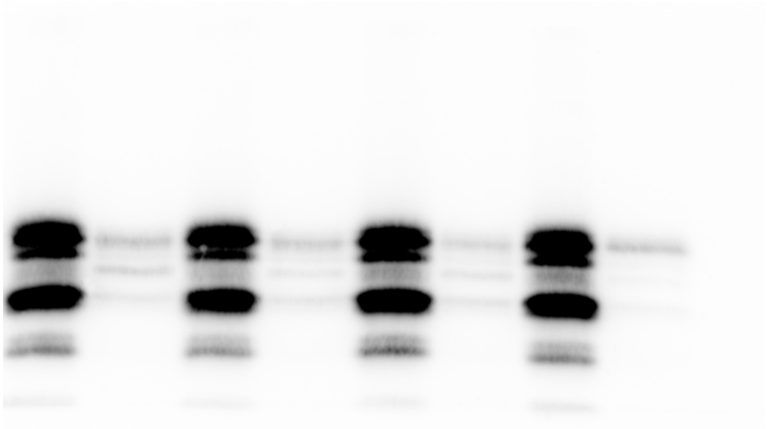

Supplementary Fig. 3a

GFP blot

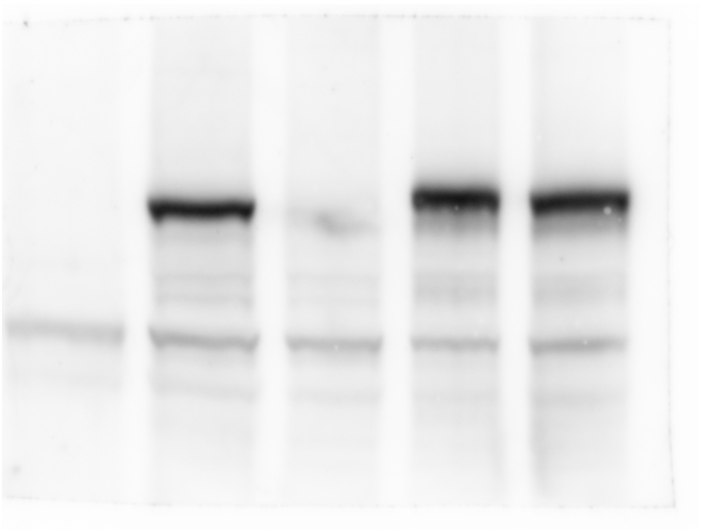

Actin blot

Supplementary Fig. 3b

AMPK blot

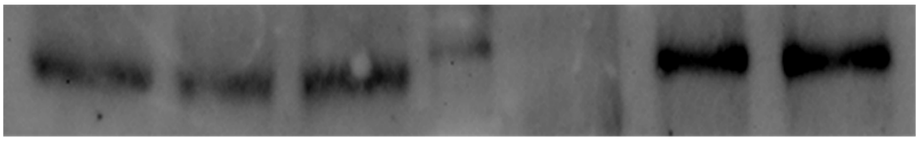

STRADα blot

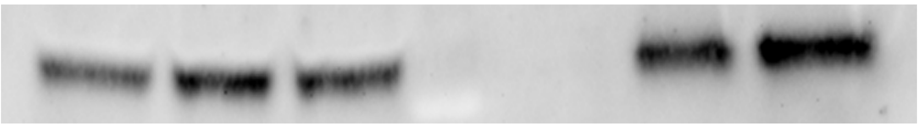

Mo25 blot IP

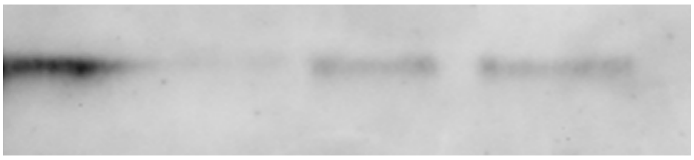

Mo25 blot Input

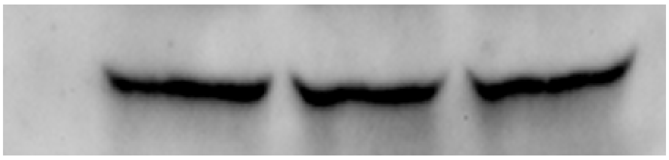

GFP blot

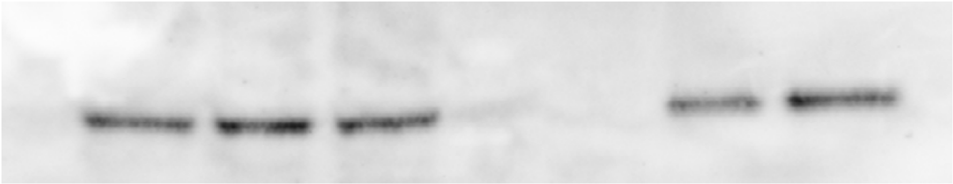

Supplementary Fig. 3d

GFP blot

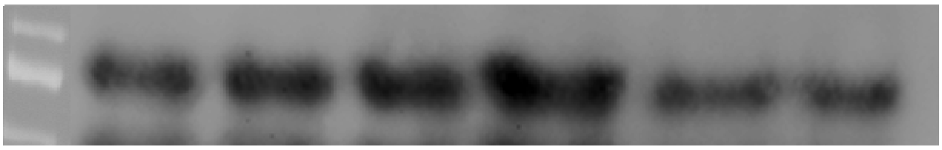

Supplementary Fig. 5b

pAMPK blot (lane 4-6)

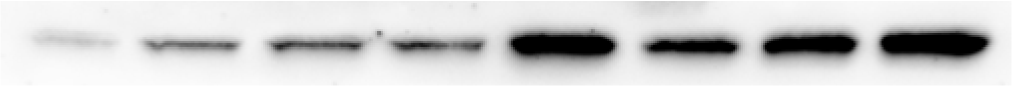

AMPK blot (lane 4-6)

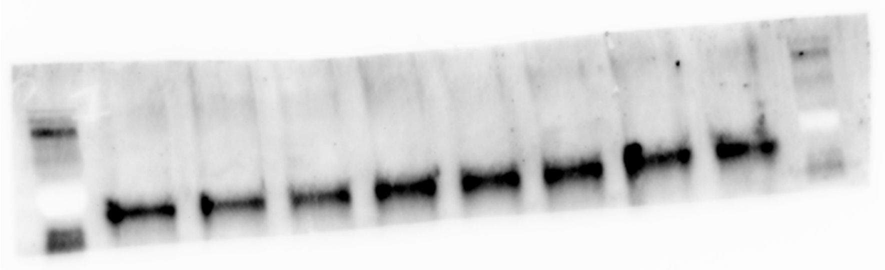

GFP blot (lane 4-6)

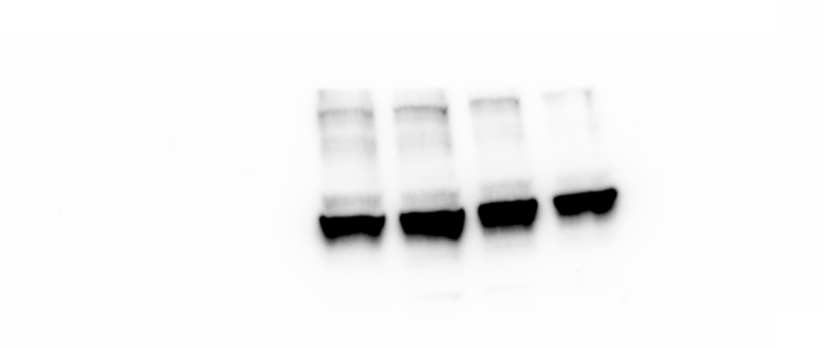

Actin blot (lane 4-6)

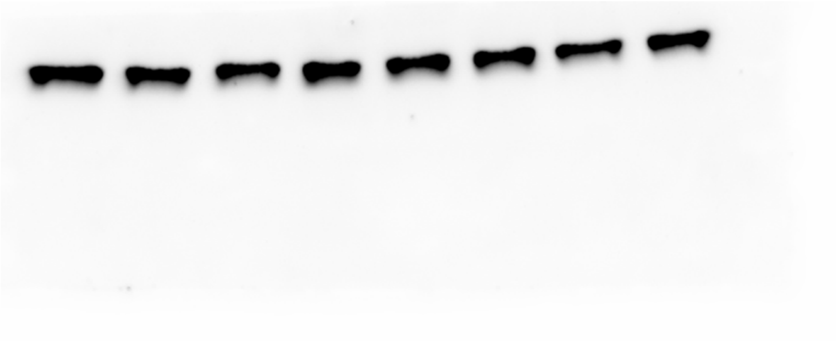

Supplementary Fig. 6a

pAMPK blot

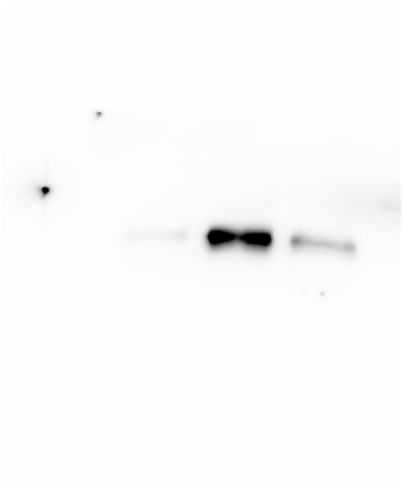

AMPK blot

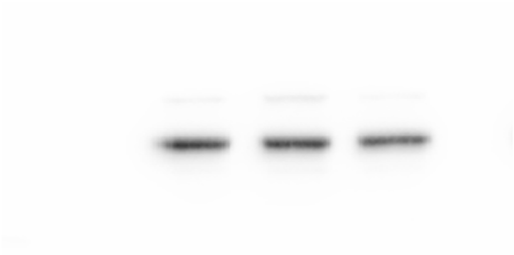

pS6K blot

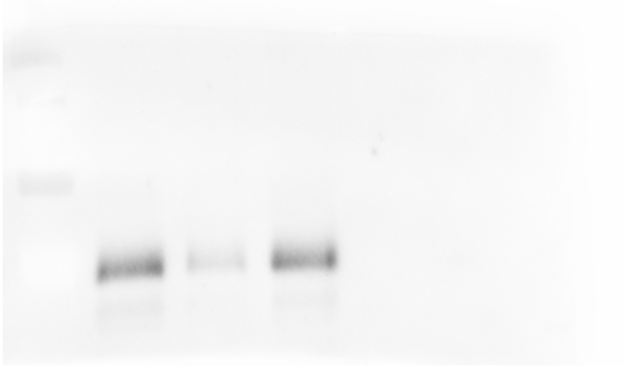

S6K blot

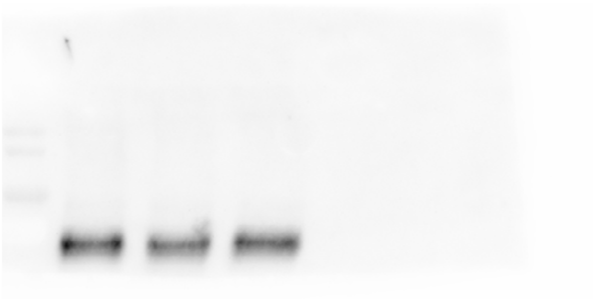

pMARK blot

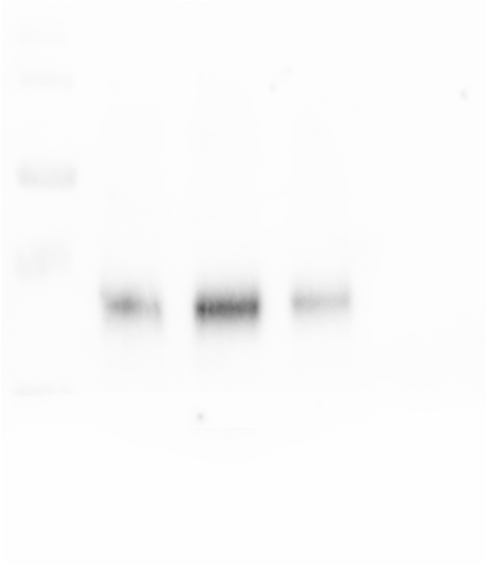

GFP blot

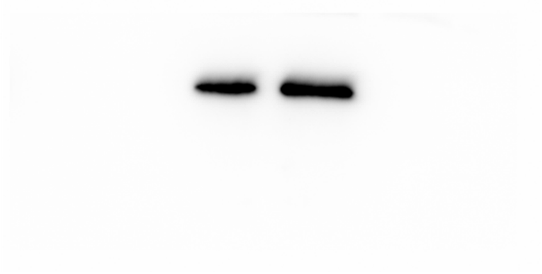

Actin blot

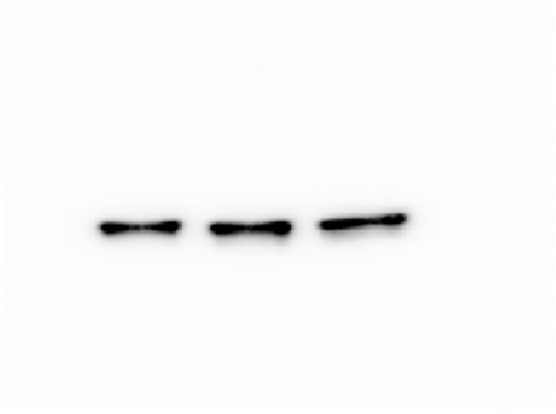

Supplementary Fig. 6b (lanes 2-5)

pAMPK blot

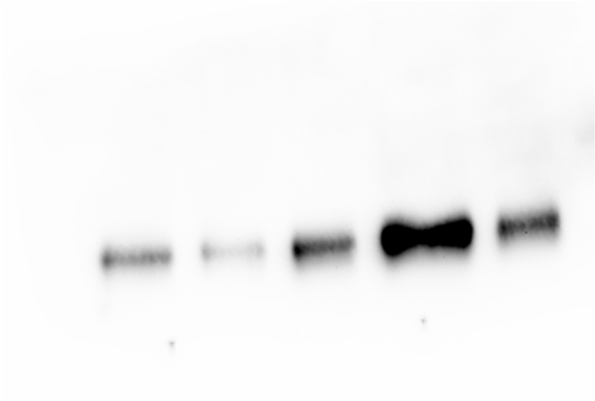

AMPK blot

pS6K blot

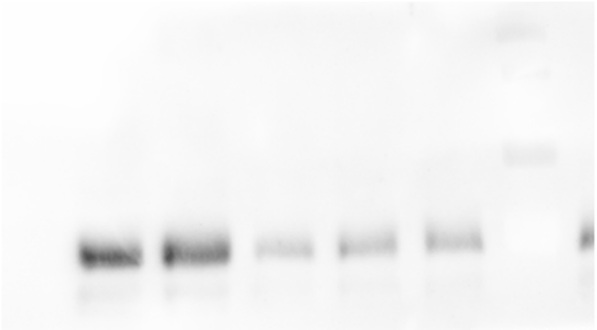

S6K blot

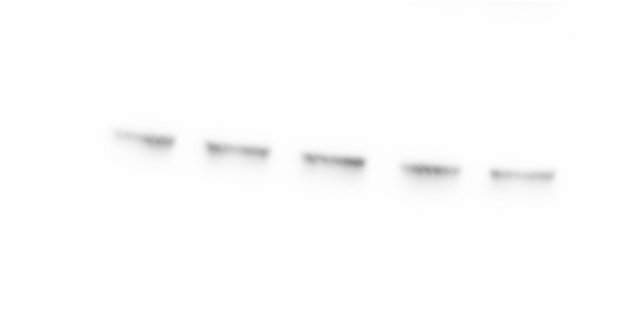

pMARK blot

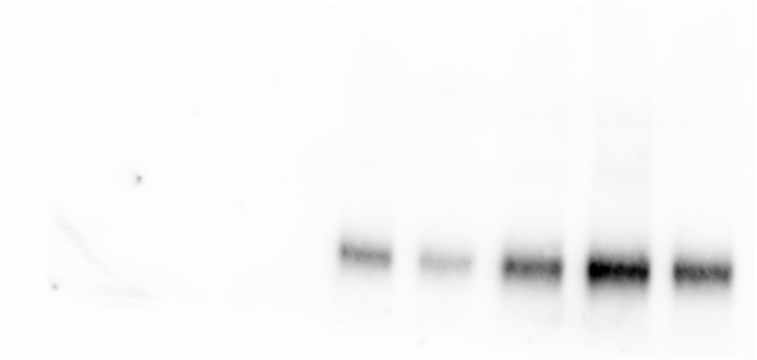

GFP blot

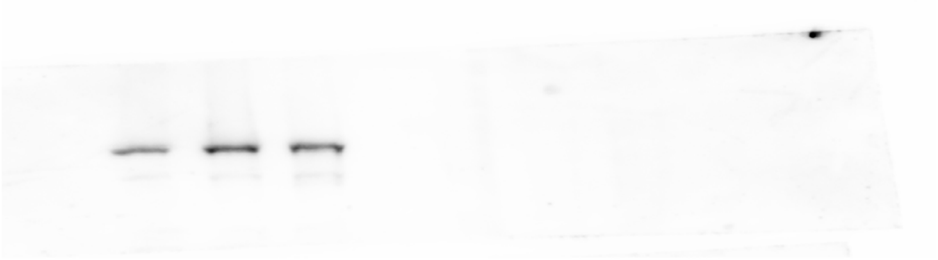

Actin blot

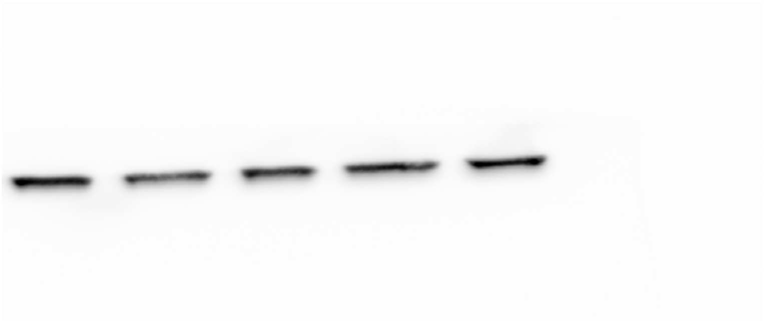

Supplement: Supplementary file 3 — Supplementary Information [file 41467_2022_28923_MOESM3_ESM.pdf]
